# Advances in Endo-Hepatology: The Role of Endoscopic Ultrasound in the Management of Portal Hypertension

**DOI:** 10.3390/diagnostics15080967

**Published:** 2025-04-10

**Authors:** Angelo Bruni, Giuseppe Dell’Anna, Jayanta Samanta, Jacopo Fanizza, Francesco Vito Mandarino, Jahnvi Dhar, Antonio Facciorusso, Vito Annese, Sara Massironi, Alberto Malesci, Giovanni Marasco, Elton Dajti, Leonardo Henry Eusebi, Giovanni Barbara, Gianfranco Donatelli, Silvio Danese, Lorenzo Fuccio

**Affiliations:** 1 IRCCS Azienda Ospedaliero-Universitaria di Bologna, Policlinico S. Orsola, 40138 Bologna, Italy; angelo.bruni4@unibo.it (A.B.); giovanni.marasco4@unibo.it (G.M.); elton.dajti2@unibo.it (E.D.); leonardo.eusebi@unibo.it (L.H.E.); giovanni.barbara@unibo.it (G.B.); lorenzo.fuccio3@unibo.it (L.F.); 2 Department of Medical and Surgical Sciences, University of Bologna, 40138 Bologna, Italy; 3 Gastroenterology and Gastrointestinal Endoscopy Unit, IRCCS San Raffaele Hospital, 20132 Milan, Italy; dellanna.giuseppe@hsr.it (G.D.); fanizza.jacopo@hsr.it (J.F.); mandarino.francesco@hsr.it (F.V.M.); sara.massironi@libero.it (S.M.); malesci.alberto@hsr.it (A.M.); 4 Gastroenterology and Gastrointestinal Endoscopy Unit, IRCCS Policlinico San Donato, 20097 Milan, Italy; vito.annese@grupposandonato.it; 5 Unité d’Endoscopie Interventionnelle, Hôpital Privé des Peupliers, 75013 Paris, France; donatelligianfranco@gmail.com; 6 Department of Gastroenterology, Post Graduate Institute of Medical Education and Research, Chandigarh 160012, India; dj_samanta@yahoo.co.in (J.S.); jahnvi3012@gmail.com (J.D.); 7 Faculty of Medicine and Surgery, Vita-Salute San Raffaele University, 20132 Milan, Italy; 8 Department of Gastroenterology and Hepatology, Punjab Institute of Liver and Biliary Sciences, Mohal 160062, India; 9 Gastroenterology Unit, Faculty of Medicine and Surgery, University of Salento, 73100 Lecce, Italy; antonio.facciorusso@unisalento.it; 10 Department of Clinical Medicine and Surgery, University of Naples “Federico II”, 80138 Naples, Italy

**Keywords:** endo-hepatology, portal hypertension, EUS-guided interventions, endoscopic ultrasound (EUS)

## Abstract

Portal hypertension (PH) is a complication of advanced liver diseases, including cirrhosis and hepatocellular carcinoma, often leading to unfavorable outcomes. Endo-hepatology, particularly endoscopic ultrasound (EUS) has revolutionized the assessment of PH. Notably, EUS-guided portal pressure gradient (EUS-PPG) enables measurement of portal and hepatic venous pressures, offering diagnostic precision for both cirrhotic and non-cirrhotic forms of PH, including porto-sinusoidal vascular disorder (PSVD). EUS-based assessment of PH in advanced liver disease can refine diagnostic workup and prognostication, supporting therapeutic decisions. Additionally, EUS-guided liver biopsy (EUS-LB) achieves high-quality histological samples with fewer complications compared to percutaneous techniques, enabling thorough evaluation of chronic liver diseases and vascular abnormalities. EUS-shear wave elastography (EUS-SWE) further refines stiffness measurements where standard imaging fails. Moreover, EUS plays a major role in controlling variceal hemorrhage, a severe PH complication. EUS-guided coil and cyanoacrylate injection for gastric varices demonstrate a great efficacy, often surpassing conventional endoscopy. Similarly, EUS-based identification and treatment of perforator vessels feeding esophageal varices reduce rebleeding risks, particularly in challenging patients. The combination of these state-of-the-art interventions supports a “one-stop strategy”, integrating variceal screening, biopsy, and portal pressure measurement within a single procedure. Despite these advancements, refinements in sedation protocols, patient selection, and cost-effectiveness data are necessary. While noninvasive tools remain central in guidelines, EUS-based methods continue to expand their role, especially in complex cases. This review summarizes the applications and impact of EUS in evaluating PH, emphasizing its importance in contemporary hepatology and its potential as a pivotal diagnostic modality in cirrhosis complicated by PH.

## 1. Introduction

In recent years, hepatology has undergone a transformative evolution driven by significant advancements in diagnostic and therapeutic technologies. A pivotal innovation within this field is the integration of endoscopic ultrasound (EUS), which has catalyzed the emergence of endo-hepatology. This specialized discipline seamlessly merges the diagnostic precision of advanced imaging with the therapeutic versatility of minimally invasive interventions, addressing the complexities of chronic liver diseases and their sequelae. The most useful techniques for assessing and stratifying the risk of clinically significant portal hypertension (CSPH) are EUS-guided techniques, including portal pressure gradient (EUS-PPG) measurement, liver biopsy (EUS-LB), and shear wave elastography (EUS-SWE), a specific form of elastometry [1,2].

These methodologies have expanded the diagnostic and therapeutic repertoire available to clinicians, enabling the precise assessment and management of hepatic parenchymal disorders, vascular pathologies, and portal hypertension. For instance, EUS-PPG offers direct quantification of portal and hepatic venous pressures, providing an accurate diagnostic alternative to the hepatic venous pressure gradient (HVPG) measurement, particularly in identifying CSPH and non-cirrhotic etiologies such as porto-sinusoidal vascular disorder (PSVD) [3]. This technique is pivotal in risk stratification and guiding management strategies for variceal hemorrhage and other complications. Equally interesting is the application of EUS-LB, which has redefined tissue acquisition in hepatology. Unlike traditional percutaneous or transjugular approaches, EUS-LB permits the safe and effective sampling of both hepatic lobes within a single session, minimizing procedural risks while maximizing diagnostic yield [4]. This innovation is particularly advantageous in patients with challenging anatomy or other contraindications to percutaneous biopsy, offering a precision approach that simultaneously allows targeting hepatic focal lesions that are not accessible through percutaneous routes.

EUS-SWE further complements the diagnostic landscape by facilitating real-time evaluation of liver stiffness, an essential parameter in the staging of fibrosis and cirrhosis. Unlike transient elastography (TE) or magnetic resonance elastography (MRE), EUS-SWE provides superior accuracy in patients with obesity or narrow intercostal spaces and can be integrated into routine EUS evaluations for seamless clinical workflow [5]. The versatility of EUS extends to therapeutic interventions, including the obliteration of gastric varices via cyanoacrylate injection or coil embolization, offering a minimally invasive alternative to conventional endoscopic or radiologic approaches [6,7]. Furthermore, the “one-stop strategy” afforded by endo-hepatology integrates variceal screening, liver stiffness assessment, biopsy, and portal pressure measurement into a single procedure, streamlining care pathways and reducing patient burden [8,9,10].

**Figure 1:**
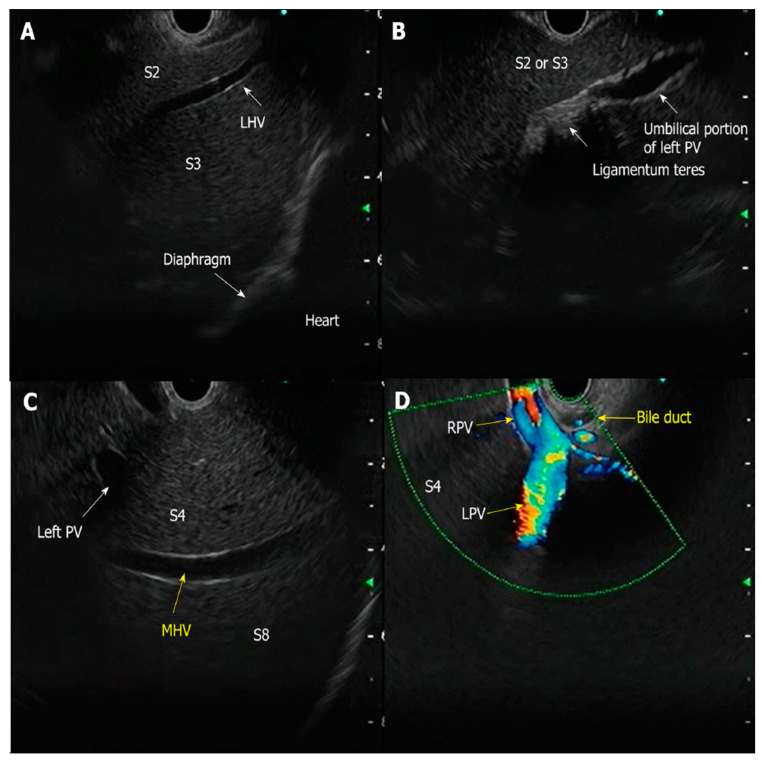
B-mode scanning of the liver using linear echoendoscope reveals (**A**) anatomy of the left lobe with S2 and S3 segments; (**B**) this image shows ligamentum teres with umbilical portion of the left portal vein (PV); (**C**) this image shows middle hepatic vein (MHV) with segments of the liver; and (**D**) this image shows anatomy of the bifurcation of portal vein (PV) from the duodenal bulb (permission has been taken from Dr. Jayanta Samanta for the publication of this image); [Abbreviations: PV: portal vein; MHV: middle hepatic vein; LHV: left hepatic vein; RPV: right portal vein; LPV: left portal vein]. The copyright of the image belongs to the authors.

Despite these advancements, which include the precise evaluation of hepatic vascular structures (Figure 1), several challenges remain. Standardization of procedural protocols, optimization of sedation techniques, and validation of cost-effectiveness are imperative to ensure broader adoption and integration of EUS-based methodologies.

Identifying a defined patient cohort that could benefit from a one-stop strategy combining the diagnostic and therapeutic capabilities of EUS represents a promising avenue in personalized medicine. Such an approach would streamline care, allowing precise risk stratification and targeted interventions within a single procedure, optimizing both clinical outcomes and resource utilization.

To address the key clinical challenge of optimizing both the diagnostic and therapeutic management of portal hypertension, we formulated a guiding question: can a “one-stop” endo-hepatology approach, centered on EUS-based modalities, improve risk stratification and interventional outcomes relative to traditional methods? In attempting to answer this question, we synthesized the available evidence, highlighted existing methodological limitations, and considered avenues for future large-scale validation studies within this rapidly evolving field of endo-hepatology. This review explores the entire spectrum of EUS applications in the context of portal hypertension, emphasizing their practical utility and the potential to reshape the contemporary framework of hepatology practice.

## 2. Materials and Methods

To provide a comprehensive and up-to-date overview of endo-hepatology, we conducted a narrative literature review using PubMed, Embase, and Scopus, from database inception to February 2025. We focused on prospective and retrospective studies, meta-analyses, and relevant guidelines that investigated the role of endoscopic ultrasound (EUS) in hepatology. Particular attention was given to EUS-guided liver biopsy (EUS-LB), shear wave elastography (EUS-SWE), and portal pressure gradient measurement (EUS-PPG). English-language publications were considered, using combinations of terms including the following: “hepatology”, “liver”, “liver tumor”, “liver stiffness”, “portal vein”, “varices”, “portal hypertension”, “liver elastography”, “liver biopsy”, “portal pressure gradient”, “endoscopic ultrasound”, and “EUS”. Additional references were identified through manual searches in Google Scholar and the Cochrane Library, and by examining the bibliographies of key reviews in the field. The literature was selectively summarized to highlight current applications, emerging techniques, and areas requiring further research in the evolving landscape of EUS-based endo-hepatology.

## 3. Endoscopic Ultrasound Guided Elastography

### 3.1. Strain Elastography

**Figure 2:**
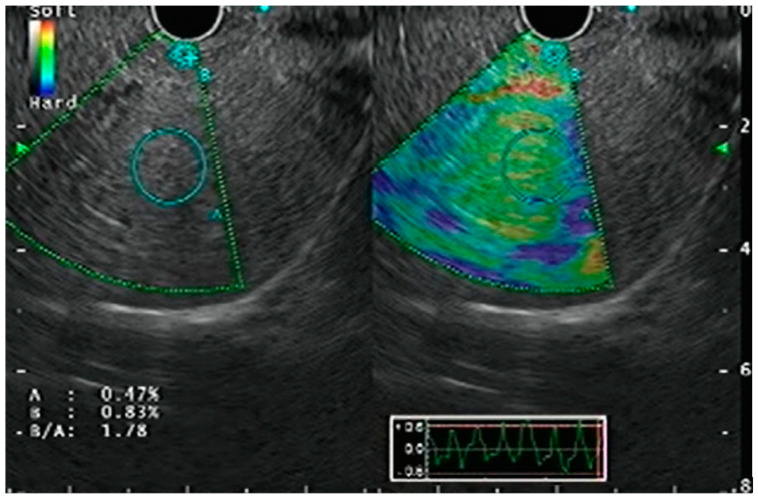
EUS-elastography of the liver parenchyma showing a strain ratio of 1.78 (permission has been taken from Dr. Jayanta Samanta for the publication of this image). The copyright of the image belongs to the authors.

Strain elastography is based on the principle that pathological processes, including cancer, inflammation, or fibrosis, alter tissue stiffness [11]. By measuring subtle tissue deformations—induced by the EUS probe’s gentle compression and augmented by vascular pulsations and respiratory movements—this technique provides real-time strain data [12]. Qualitative strain elastography superimposes a color-coded map (dark blue for stiff tissues, red for softer regions) on the B-mode image (Figure 2) [13]. Accurate endoscope positioning with consistent pressure application improves imaging quality, and frame averaging minimizes operator bias. Although initially used primarily for evaluating pancreatic lesions and lymph nodes, strain elastography has also been applied for hepatic stiffness assessment in the context of EUS, offering both qualitative and quantitative insights into liver disease. Quantitative strain elastography further refines this evaluation through strain ratio and strain histogram analyses, comparing the stiffness of a suspicious lesion or parenchymal region to that of surrounding reference tissue [14,15,16,17]. In instances where color mapping is indeterminate, these quantitative metrics furnish objective support for real-time clinical decision-making, thus making strain elastography a potentially valuable tool for both focal and diffuse liver stiffness assessment via EUS.

### 3.2. Shear Wave Elastography

EUS-based elastometry, particularly EUS-SWE, has emerged as a valuable tool for assessing hepatic fibrosis in both parenchymal diseases and focal lesions [18]. Although current evidence is primarily derived from pilot or observational studies involving over 200 patients, these data suggest that EUS-SWE can differentiate advanced liver fibrosis (≥F3) with AUROC values ranging from 0.89 to 0.93 [5,19,20]. In a cohort of 42 patients, the cross-validated AUROC for detecting cirrhosis reached 0.90, with successful measurements even in individuals with a BMI exceeding 30 kg/m^2^ [20].

EUS-SWE outperformed vibration-controlled transient elastography (VCTE) in obese populations, achieving AUROC values of 0.78–0.89 in patients with a median BMI near 40 kg/m^2^, while VCTE accuracy dropped below 0.70 [21]. Additionally, VCTE was unreliable in 19% of cases due to high skin-to-liver distances and poor signal transmission leveraging the endoscopic probe’s proximity to hepatic parenchyma to bypass the thick anterior abdominal wall.

EUS-SWE, performed via a linear echoendoscope in the proximal stomach or duodenum, involves selecting a 10–15 mm region of interest at least 1 cm below the liver capsule while avoiding large vessels. Ten acquisitions per site are recommended, with median kilopascal values and interquartile ranges ideally <30% [20].

Different studies propose distinct cutoff values depending on the goal of maximizing sensitivity or specificity. In one investigation, a cutoff of approximately 7.5 kPa yielded 90% sensitivity for excluding significant fibrosis (≥F2), whereas increasing that threshold to 10.2 kPa still produced about 90% specificity for advanced disease [22,23]. Overall, an interesting 2023 study compared EUS-SWE, VCTE, and histology in 42 patients with unreliable noninvasive testing. While VCTE was unreliable in 19% of cases, EUS-SWE succeeded generally. AUROCs for advanced fibrosis were similar, respectively: VCTE (0.87), left-lobe EUS-SWE (0.80), and right-lobe EUS-SWE (0.78). For cirrhosis, left-lobe EUS-SWE achieved the highest AUROC (0.96), aligning closely with histology, confirming its reliability for fibrosis staging [20]. Supporting this, a successive prospective trial reported a >90% success rate in distinguishing early-stage from advanced fibrosis when EUS-SWE measurements were combined with histology-based data [24].

By allowing direct, real-time visualization of the liver’s parenchyma, EUS-based elastometry can potentially mitigate sampling failures observed in transabdominal approaches as summarized in Table 1.

## 4. Liver Biopsy

Liver biopsies remain the gold standard for diagnosing and staging liver disease and are especially relevant in cases of presinusoidal portal hypertension or in patients with non-cirrhotic etiologies—such as porto-sinusoidal vascular disorder (PSVD)—where histologic confirmation is essential for accurate diagnosis. According to AASLD guidelines, a successful biopsy is defined by its width, length, and the number of complete portal tracts (CPTs), requiring a specimen at least 20 mm long with 11 or more CPTs [25].

**Figure 3:**
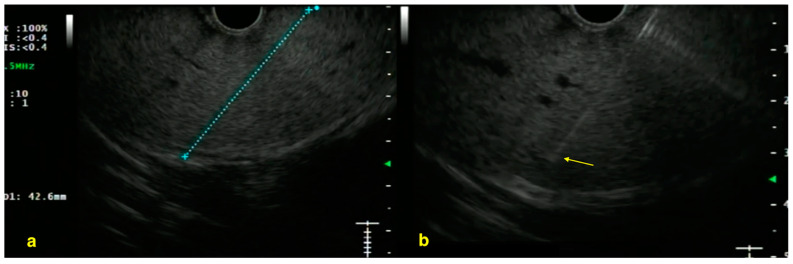
EUS-guided parenchymal liver biopsy (**a**) assessment of a vessel-free trajectory in the left lobe before puncture; and (**b**) left lobe liver biopsy with 19-G EUS-FNB needle (yellow arrow—the tip of the needle visualized) (permission has been taken from Dr. Jayanta Samanta for the publication of this image). The copyright of the image belongs to the authors.

EUS-guided liver biopsy has proven to be a reliable diagnostic technique, consistently achieving a diagnostic yield exceeding 90% in terms of complete portal tracts and specimen length across multiple trials, demonstrating its non-inferiority to percutaneous and transjugular approaches [26]. In a large meta-analysis of 23 studies involving over 1300 patients, EUS-LB achieved a pooled diagnostic adequacy of 93.9%, which rose to nearly 95% when the other generation Tru-Cut needle data were excluded [27]. These values are comparable to common transhepatic LB and suggest that EUS guidance, particularly with dedicated fine-needle biopsy (FNB) devices, is well suited to obtain high-quality cores for histopathological assessment (Figure 3). Multiple prospective trials have demonstrated that EUS-LB is associated with fewer adverse effects, such as reduced pain, and offers high diagnostic accuracy with a strong correlation to histologic staging, also in NASH patients [26,28].

One major advantage of EUS over transhepatic or transjugular sampling is its real-time visualization of the liver parenchyma, enabling precise needle placement while avoiding large vessels. This is particularly beneficial for borderline lesions poorly characterized on imaging or difficult to approach with other methods. Bilateral lobe sampling reduces errors in heterogeneous conditions like NASH, often achieving 15–20 complete portal tracts and improving fibrosis or inflammatory staging accuracy. Studies comparing left-lobe (stomach-rout) and right-lobe (duodenum-rout) approaches report no significant yield differences [4,29].

The choice of needle and the technique used are crucial for optimizing histologic yield. While early studies with 19-gauge fine-needle aspiration (FNA) needles faced limitations such as fragmented cores and lower portal tract counts, the introduction of advanced Franseen and Fork-tip FNB systems has markedly improved tissue acquisition. In cohorts undergoing biopsy with a 19-gauge Franseen tip needle, intact core lengths have averaged well above 20 mm, with PTCs exceeding 10 in more than 80% of patients [30,31].

A recent trial reported that the fork-tip needle achieved ≥11 portal tracts in up to 85% of cases—surpassing other FNA needles, which had lower rates of complete portal tract retrieval (*p* < 0.01)—and that wet-suction and heparin-primed techniques further improved core integrity by reducing blood contamination [31,32,33,34].

However, the recent “dynamic suction” approach, combining wet suction with precise heparin priming and controlled suction timing, has demonstrated superior results, with an average core length of 6.45 cm and over 25 complete portal tracts per sample [35]. These results significantly exceed the adequacy criteria set by the AASLD, highlighting the technique’s potential to optimize liver tissue acquisition. When juxtaposed with percutaneous biopsy, EUS-guided liver sampling confers several practical benefits, including shorter post-procedure recovery and less procedure-related pain, likely due to avoidance of the Glisson capsule puncture as summarized in Table 2 [36,37,38,39,40,41]. Despite slightly longer procedure times (mean of 5–7 min) compared to percutaneous biopsy, the overall experience is well tolerated [42].

However, a 2021 randomized trial by Bang et al. provides a counterpoint: in consecutive patients, transabdominal ultrasound-guided percutaneous biopsy produced “optimal” specimens (≥25 mm length and ≥11 complete portal tracts) in 57.9% of cases versus 23.8% with EUS-guided biopsy (*p* = 0.028), and was cheaper (US$1824 vs. US$3240, *p* < 0.001) [43]. Although, the percutaneous biopsy caused more immediate postprocedural pain. Notably, the trial used a stringent adequacy definition that may be challenging in everyday practice, whereas EUS-guided biopsy enables bilobar sampling and concurrent endoscopic procedures.

In addition, cost analyses suggest that, when performed in conjunction with indicated endoscopic examinations (such as screening endoscopy for varices), EUS-liver biopsy is cost-effective and may expedite comprehensive management [44,45].

## 5. Portal Pressure Gradient Measurement

The hepatic venous pressure gradient (HVPG) is a critical parameter for evaluating clinically significant portal hypertension (CSPH). An HVPG value exceeding 10 mmHg indicates CSPH and associated risk of variceal development, while levels above 12 mmHg signify an elevated bleeding risk. However, this method may not detect non-cirrhotic forms of portal hypertension, like PSVD, and involves exposure to radiation [46,47].

Moreover, HVPG is deemed unsuitable for assessing presinusoidal portal hypertension, particularly in disorders such as primary sclerosing cholangitis, primary biliary cholangitis, polycystic liver disease, and malignancies [48,49]. Additionally, its accuracy in evaluating portal hypertension in MAFLD, currently the foremost cause of cirrhosis and liver transplantation, is hindered by the unique pathophysiological mechanisms underlying this condition. Conversely, EUS-PPG has gained significant attention as a direct and innovative technique for portal hypertension evaluation, offering a distinctive combination of benefits and inherent limitations, as summarized in Table 3.

In a porcine model published by Huang et al., a strong correlation (r = 0.99) was observed for the first time between EUS-PPG and the standard HVPG, indicating the feasibility of the approach until 2016 [50]. In the human experience reported by the same group, patients underwent EUS-PPG measurement with a 100% technical success rate, and none experienced serious adverse events [51].

**Figure 4:**
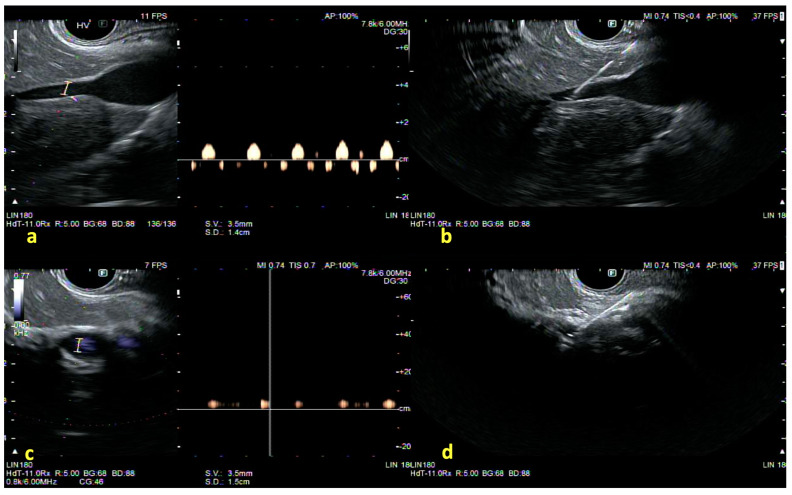
EUS-guided portal pressure measurement: (**a**) identification of the hepatic vein (HV) using power Doppler, which revealed a multi-phasic waveform; (**b**) then, puncture of hepatic vein (HV) with a 25-G EUS-FNA needle; (**c**) identification of the portal vein (PV) using power Doppler, which revealed a monophasic waveform; and (**d**) finally, puncture of the portal vein with a 25-G EUS-FNA needle (permission has been taken from Dr. Jayanta Samanta for the publication of this image). The copyright of the image belongs to the authors.

The procedure generally uses a 25 G needle (Cook EchoTip Insight), heparinized saline, and a digital manometer zeroed at the mid-axillary line [52]. After B-mode and Doppler imaging identify a branch of the hepatic vein, typically the middle hepatic vein, the needle is advanced transgastrically through a small segment of hepatic parenchyma (Figure 4). Three readings are obtained once the manometer stabilizes, with a flush of 0.5–1.0 mL of saline before each measurement [53]. Each measurement lasts around 60 s, and at least three measurements per vessel are recommended, discarding any outlier readings potentially caused by clot formation or contact with the vessel wall [54]. Verifying that the final PPG value aligns with endoscopic findings (such as varices or portal hypertensive gastropathy) helps rule out measurement errors or unrecognized confounding factors like right-sided heart failure. The average of these readings is recorded as the hepatic vein pressure. Subsequently, an intrahepatic branch of the portal vein is targeted under Doppler guidance, and the same steps are repeated to acquire the portal pressure. The difference between the mean portal and hepatic vein pressures constitutes the PPG value [55].

In a recent trial, EUS-PPG achieved success rates exceeding 90%, with mild, transient pain as the most common adverse event [56]. These findings were corroborated by a 2023 meta-analysis by Dhindsa et al., which included eight cohort studies (178 total patients) and demonstrated a pooled technical success rate of 94.6% and an overall complication rate of 10.9%, primarily mild pain or minor bleeding [57]. Given that cirrhotic patients often present with thrombocytopenia, standardized procedural protocols are crucial for reproducibility. Notably, many studies excluded patients with platelet counts <50,000/μL or large-volume ascites, thereby limiting the current evidence base in advanced cirrhosis. Furthermore, EUS-PPG has demonstrated diagnostic utility in patients with PSVD. Zhang et al. reported its use in nine individuals with sinusoidal obstruction syndrome, finding mean PPG values of 18.07 ± 4.30 mmHg, which were comparable to HVPG measurements (18.82 ± 3.43 mmHg; *p* = 0.15), with a strong correlation between the two methods (r = 0.92) [51].

Expert consensus advises performing HVPG under minimal to no sedation, limiting doses to a maximum of 0.02 mg/kg of midazolam, to preserve measurement accuracy [54]. In contrast, all EUS-PPG studies published to date have utilized moderate to deep sedation, which is known to affect portal pressure readings and may lead to misclassification of portal hypertension severity. For example, Hajifathalian et al. found that performing EUS-PPG under general anesthesia with endotracheal intubation and neuromuscular blockade resulted in significant variability in venous pressure measurements, with 9 out of 24 patients exhibiting ≥10% variability, which is considered clinically meaningful [58]. Such fluctuations highlight the potential for sedation to compromise the reliability of PPG readings, particularly as HVPG differences as small as >1 mm Hg are clinically very significant. Given that EUS-PPG cannot be performed without sedation, it is imperative to develop standardized protocols that consider sedation’s impact and ensure measurements closely approximate awake values.

## 6. EUS-Guided IPSS and Portal Blood Sampling

EUS-guided intrahepatic portosystemic shunt (IPSS) and Portal Blood Sampling have emerged as two notable frontiers in endohepatology. They are broadening the scope of minimally invasive interventions for portal hypertension and offering unprecedented insights into gut-liver pathophysiology.

The IPSS under EUS guidance has been explored primarily in large-animal studies, where a transgastric approach allows the creation of a portosystemic bypass by accessing an intrahepatic branch of the portal vein and a branch of the hepatic vein (often the left hepatic vein) [59]. This technique was subsequently reproduced in an additional animal cohort in 2016 using lumen-apposing metal stents (LAMS), resulting in measurable decreases in portal pressures comparable to those achieved by a transjugular intrahepatic portosystemic shunt (TIPS) [60].

Preclinical reports have shown promising results regarding portal decompression, but key questions regarding stent durability, bleeding risk within the hepatic parenchyma, and optimal patient selection remain unresolved.

A novel EUS-guided technique involves the puncture of the portal vein to directly sample portal blood. Historically, this required surgical intervention or an existing TIPS stent, posing significant limitations. By attaching a Vacutainer Luer-lock adaptor to the EUS needle handle, rather than using manual syringe aspiration, hemolysis is greatly reduced and sample volumes exceeding 2.5 mL can be obtained [61].

By leveraging EUS-based visualization, clinicians and researchers can interrogate microbial products, metabolic signatures, or immunologic factors unique to the gut–liver axis in real-time, with early studies suggesting it is technically feasible and acceptably safe [62]. This approach may prove especially valuable for examining free-circulating tumor DNA in patients with colon neoplasms, potentially refining metastatic risk stratification by detecting early dissemination of malignant cells or their DNA via the portal route. However, no standardized protocols yet exist for needle specifications, sample volumes, or specific biomarkers, and validation studies are needed to ascertain procedural reproducibility and clinical impact.

## 7. Endoscopic Ultrasound-Guided Vascular Interventions

EUS-guided approaches have become an essential pillar in the therapeutic management of gastric varices (GV), which represent up to 10% to 20% of variceal bleeding in cirrhotic patients. Although less frequent than esophageal varices (EV) overall, bleeding from GV can have higher transfusion requirements and mortality rates—often exceeding 30% in severe presentations—compared to bleeding from EV [63]. Multiple observational studies and controlled trials indicate that EUS-guided techniques may offer distinct advantages, including a reduction in recurrent hemorrhage, compared to direct endoscopic glue injection (EGI) [64]. EUS guidance leverages a real-time Doppler assessment of both the varix and its feeding vessels, enabling the clinician to visualize flow patterns and variceal anatomy. This contrasts with conventional endoscopic injection approaches, wherein the operator may only partially identify or target the varix, especially if visibility is impaired by active bleeding or anatomic constraints [65]. In a single-center experience, EUS enabled clear delineation of the afferent vessel in 88% of patients with large GV, whereas transendoscopic color Doppler alone identified such vessels only 52% of the time [66]. This superior visualization under EUS guidance can translate into fewer incomplete obliterations, a lower volume of sclerosant or cyanoacrylate (CYA) required, and a potentially reduced risk of systemic embolization.

Data from a 2020 meta-analysis [67] encompassed 18 cohort studies (604 patients) of EUS-guided therapy and showed a pooled variceal obliteration rate of 84% and an overall rebleeding rate of 11.6%. By comparison, direct endoscopic CYA injection exhibited a pooled rebleeding rate of approximately 17%. A more recent multicenter series by Samanta et al. [68], which included 276 patients, matched 58 EUS-guided coil/glue recipients with 218 controls who underwent standard CYA injection. Within a 6-month follow-up, the EUS group experienced a 9% to 10% rebleeding rate, compared to nearly 25% in the EGI cohort (*p* < 0.05), with a technical success exceeding 90% for both techniques. A randomized controlled trial by Lôbo et al. [69] further demonstrated the safety profile of EUS-guided coil + glue injection. Among 16 patients treated with EUS guidance vs. 56 patients receiving the conventional endoscopic injection, variceal obliteration was achieved in 81% vs. 63%, respectively (*p* = 0.02), with a lower 1-year rebleeding rate in the EUS group (12.5% vs. 29.0%). Another trial found that up to 4% to 5% of patients undergoing either standard or EUS-guided injections experienced clinically evident systemic embolization, but the majority of these events were subclinical and detected only on screening imaging [70].

**Figure 5:**
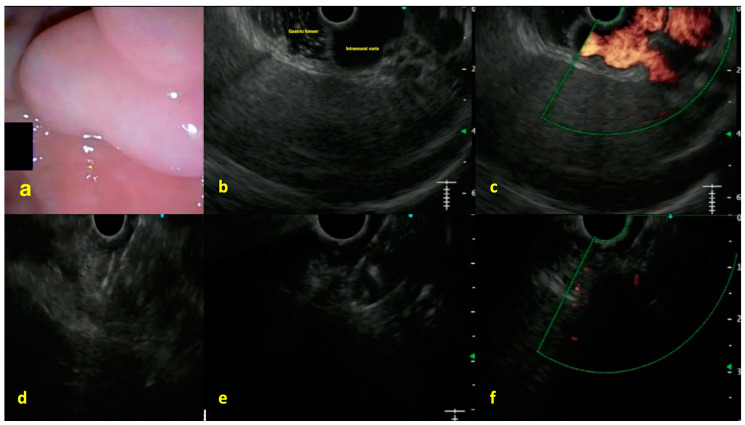
EUS-guided angioembolization of the gastric fundal varices (**a**) endoscopic view of the fundus reveals large varix (GOV2F2); (**b**) linear EUS examination was performed for variceal assessment by instillation of water in the fundus of the stomach and it revealed a large intramural varix (submucosal component); (**c**) Doppler assessment using EUS positive flow in varix; (**d**) varix was punctured using a 19-G EUS-FNA needle and Nester coils were deployed under EUS vision; (**e**) after the varix was packed with the sufficient number of coils, cyanoacrylate glue was injected under EUS-vision leading to the formation of the coil-glue cast; and (**f**) confirmation of complete variceal obliteration using color Doppler assessment (no positive flow noted) (permission has been taken from Dr. Jayanta Samanta for the publication of this image). The copyright of the image belongs to the authors.

An interesting aspect of EUS-based interventions is the combination of coil deployment and cyanoacrylate injection (Figure 5). The coil acts as a scaffold and reduces blood flow, while the glue polymerizes around it, reducing the total amount of CYA required by 30% to 60% in some series. In large U.S. experiences spanning more than six years, reported by Bhat et al. [71] and extended by Bazarbashi et al. [72], technical success rates of 95% to 100% were observed, with 6% to 15% rebleeding rates at 12 months.

However, pulmonary or systemic glue embolization remains a primary concern in CYA-based treatments. Most large studies indicate a clinically symptomatic embolization rate of 1% to 3%, with asymptomatic rates higher (5% to 10%) on routine imaging [73,74]. In a propensity-score matched analysis of 58 EUS-treated patients vs. 218 direct endoscopic injection controls, Samanta et al. [68] found a non-significant trend toward fewer embolic events in the EUS group (2% vs. 5%, *p* > 0.05), potentially because of lower glue volumes. However, meta-analyses and trials have not conclusively demonstrated a statistically significant difference in pulmonary embolism risk between EUS-based coil + CYA and direct injection therapy, likely due to heterogeneous definitions and small numbers of events [67,68,75].

An additional major benefit of EUS guidance is the improved reduction in the reintervention rate. A 2023 retrospective study involving 57 patients (21 EUS-coil + CYA vs. 36 endoscopic CYA alone) showed the EUS approach had a significantly lower 12-month reintervention rate (10% vs. 31%, *p* < 0.01) [73]. Despite these promising results, overall mortality remains influenced primarily by the severity of underlying liver disease. All-cause mortality at 6 to 12 months is frequently reported in the 15% to 25% range, most attributed to advanced cirrhosis, infection, or hepatocellular carcinoma [67,69,72].

## 8. Additional Role of EUS-Guided Ablation in Liver Tumors

EUS-guided tumor ablation has recently emerged as a minimally invasive strategy for selected patients with primary or metastatic liver lesions, particularly those in the left or caudate lobe or deep subcapsular locations not easily accessible via percutaneous routes. Several case reports have documented the application of EUS-guided radiofrequency ablation (RFA) in hepatocellular carcinoma (HCC), showing that it can achieve sustained local control in patients who would otherwise be poor candidates for curative treatment. In one of the earliest reports, Attili et al. demonstrated complete tumor disappearance in a 25 mm HCC located in segment III, with no reported adverse events and durable tumor-free imaging at one month [76]. Subsequent observations by de Nucci et al. [77] and Katrevula et al. [78] confirmed the feasibility of EUS-RFA for subdiaphragmatic HCCs in segment II-III and the caudate lobe, respectively, with partial or complete ablation in short-term follow-up. Although high-risk comorbidities or challenging tumor positions can preclude percutaneous or surgical therapy, EUS guidance—owing to the echoendoscope’s proximity to the left and caudate lobe—may allow real-time monitoring of ablation and precise needle placement, thereby reducing the risk of complications such as pleural effusion or thermal injury to surrounding structures. In the largest experiences published to date, EUS-RFA was associated with acceptable safety profiles and only transient biochemical alterations, without major adverse events such as liver abscess or hemorrhage [79,80]. While further large-scale studies and longer follow-up are required, EUS-guided ablation appears to complement the diagnostic and interventional capabilities of endo-hepatology, offering a new therapeutic horizon for a select group of patients with HCC who are otherwise difficult to treat.

## 9. Conclusions and Future Perspectives

Ongoing developments in endo-hepatology are poised to expand the diagnostic and therapeutic capabilities of EUS-based liver interventions. Although current data already support the feasibility and accuracy of EUS-guided liver biopsy, elastography, and portosystemic pressure gradient measurement, several key areas stand out for future exploration and advancement.

First, integrating artificial intelligence (AI) into EUS image acquisition, interpretation, and elastography analysis offers the potential to improve diagnostic precision markedly. Preliminary pilot studies suggest that machine learning applications could guide optimal needle trajectories in EUS-guided liver biopsy, lessen artifacts, and reduce variability in portosystemic pressure measurements [81,82].

Second, EUS-guided portal venous blood sampling can be relevant to the study of portal hypertension by allowing direct access to the portal circulation. This approach, while potentially useful for detecting circulating tumor biomarkers in patients at higher risk for hepatic or extrahepatic metastases, also enables advanced analyses of the gut-liver axis—such as metabolomic or microbiome profiling—thereby offering insights into disease progression, therapeutic response, and the development of complications in conditions like portal vein thrombosis.

Third, contrast-enhanced EUS (CE-EUS) holds promise for refining the assessment of portal hypertension by measuring hepatic vein arrival time (HVAT), an indicator inversely correlated with the severity of liver dysfunction and portal hypertension [83,84,85]. HVAT is the time taken for the microbubble contrast agent to arrive at the hepatic vein after injection. Although currently supported by limited data, the ability to evaluate HVAT through CE-EUS could expand the multiparametric approach to PH evaluation, particularly in patients with compensated cirrhosis [86,87]. Further research is needed to define clear protocols for HVAT measurement in CE-EUS and to determine whether it can serve as a reliable predictor of CSPH.

Finally, clinical workflows are likely to identify patients who derive maximal benefit from a one-stop approach integrating EUS-based diagnostics and interventions. However, one of the principal limitations of EUS-based techniques is their marked operator dependency; achieving reliable, reproducible results requires significant expertise, underscoring the need for systematic training protocols and clearly defined learning curves. In addition, cost-effectiveness data remain scarce, and large-scale comparative trials are needed to determine whether the benefits of reduced complications and comprehensive one-stop strategies truly justify the financial implications of more advanced equipment and longer procedure times. From a clinical standpoint, barriers to widespread implementation persist, including limited access to EUS technology, the potential need for deep sedation, and the absence of standardized protocols across different centers.

Another overarching challenge is the limited availability of robust, large-scale validation data beyond pilot or single-center studies. However, existing reports show promising outcomes; multicenter randomized controlled trials are essential to confirm diagnostic accuracy, refine technical details, and establish clinical practice guidelines tailored to diverse patient populations.

## Figures and Tables

**Table 1:** Comparative analysis of EUS-SWE studies for liver fibrosis assessment.

Authors, Year	Study Design	Population	Methods	Key Results
AbiMansour et al., 2025 [5]	Prospective cohort	A total of 199 patients, 25 with advanced liver disease (≥F3).	EUS-SWE on both lobes (10 readings each); correlated with ≥F3 or MRE. General anesthesia vs. sedation for reliability.	ALD patients had higher stiffness (*p* < 0.001). Right lobe AUROC = 0.80; left = 0.73. General anesthesia improved reliability. Left-lobe EUS-SWE correlated with MRE.
Kohli et al., 2023 [20]	Prospective cohort	A total of 42 suspected NAFLD patients. VCTE was unreliable in ~19%.	EUS-SWE on both lobes (10 readings each). Biopsy used as reference. Cutoffs (Youden’s index).	For ≥F3: VCTE AUROC = 0.87 vs. EUS-SWE 0.80 (left), 0.78 (right). EUS-SWE succeeded in VCTE-failed cases.
Wang et al., 2024 [21]	Prospective cohort	A total of 62 obese MASLD patients (BMI ≥ 30).	EUS-SWE (10 measurements); biopsies by EUS or surgery/IR. FIB-4, VCTE for comparison.	EUS-SWE outperformed FIB-4 for ≥F2 (AUROC = 0.87 vs. 0.61) and ≥F3 (0.93 vs. 0.63). Exceeded VCTE in advanced fibrosis.
Diehl et al., 2025 [24]	Prospective cohort	A total of 52 patients with abnormal LFTs were referred for EUS LB. Mean BMI was 35.1.	VCTE before EUS. EUS-SWE on both lobes (10 readings each). 19G Franseen LB. Compared the reproducibility of left vs. right lobe and correlation with biopsy staging.	Right-lobe EUS-SWE strongly correlated (r = 0.57) vs. left-lobe (0.37). EUS-SWE ~VCTE for advanced fibrosis accuracy; right side preferred for reproducibility.

ALD: Advanced liver disease; BMI: Body Mass Index; FIB-4: fibrosis-4 index; IR: Interventional Radiology; LB: liver biopsy; LFTs: liver function tests; MASLD: Metabolic Dysfunction-Associated Steatotic liver disease; MRE: magnetic resonance elastography; VCTE: vibration-controlled transient elastography.

**Table 2:** Comparative analysis of endoscopic ultrasound-guided and percutaneous liver biopsy.

Parameter	EUS-Guided Liver Biopsy	Percutaneous Liver Biopsy
Needle Gauge and Design	Commonly uses 19 G FNB (Franseen-tip, Fork-tip) or 19 G FNA needles Typically 1–2 passes per lobe, guided by macroscopic inspection	Generally, 16–18 G cutting needles (e.g., Menghini or Tru-Cut) Often one pass (two if needed for adequacy)
Specimen Length	Mean around 20–40 mm in most series Some randomized data report lengths up to ~30–40 mm with a 19 G FNB plus wet-suction approach	Typically ~25–30 mm (or more) with a 16 G needle
Number of Complete Portal Tracts	Typically 8–20 CPTs or more using a 19 G Franseen needle	Often 10–15 CPTs or more with a 16 G needle
Diagnostic Yield	A ~90–95% in prospective cohorts	A ~92–97% in most studies
Bilobar Sampling	Can biopsy both right and left lobes in one session Especially useful in conditions with heterogeneous involvement	Typically restricted to the right lobe unless extra passes
Adverse Event Rate	Overall complication rate ~2–10% Significant bleeding or hemoperitoneum ~1–2% Mortality is rare (case reports)	Complications ~2–5% Pain, subcapsular hematomas, occasional hemothorax, or bile leak Mortality ~0.01–0.1% in large series
Sedation and Procedure Time	Moderate-to-deep sedation or general anesthesia Typically >15 min	Local anesthesia ± mild sedation Usually 10–20 min
Contraindications	Coagulopathy, inability to tolerate sedation, large gastric varices, and massive ascites Relative contraindications: certain post-surgical anatomies (e.g., Roux-en-Y)	Coagulopathy, difficult ascites, infection at the biopsy site Overlying bowel or lung may limit safe access
When to Prefer	If concurrent EUS-based interventions are indicated If percutaneous or transjugular access is contraindicated If bilobar sampling is needed	Standard approach when only liver tissue is required and the anatomy is favorable Ideal for easily accessible right-lobe lesions under normal coagulation

EUS: Endoscopic ultrasound, LB: liver biopsy, EUS-LB: endoscopic ultrasound–guided liver biopsy, FNB: fine-needle biopsy, FNA: fine-needle aspiration, CPT: complete portal tracts, G: gauge, mm: millimeter.

**Table 3:** Comparative analysis of HVPG and EUS-PPG for portal hypertension evaluation.

Aspect	HVPG	EUS-PPG
Portal pressure measurement	Indirect (via hepatic vein catheterization), measures hepatic venous pressure gradient (HVPG)	Direct (via endoscopic ultrasound-guided puncture) measures the portal pressure gradient (PPG)
Procedure type	Angiography	Endoscopy
Required equipment	Dedicated X-ray machine, contrast agents	Conventional EUS platform, fine needle for puncture
Types of portal hypertension assessed	Sinusoidal	Sinusoidal and presinusoidal
Accuracy and reproducibility	Highly validated, reproducible with inter-observer variability < 5%	Limited validation studies
Contraindications	Allergy to contrast, severe coagulopathy (platelets < 20 × 10⁹/L or PT < 30%)	Coagulopathy (platelets < 50 × 10⁹/L or PT < 50%), contraindications for upper GI endoscopy, altered anatomy
Additional procedures possible	Transjugular liver biopsy, cardiopulmonary pressure assessment	All types of additional endoscopic interventions (e.g., variceal assessment and treatment, mucosal biopsies, FNA/B of lesions, SWE measurement)
Patient sedation	Local anesthesia or mild sedation	Conscious sedation
Procedure time	30–60 min	30–60 min
Safety profile	Invasive, rare complications include bleeding, hematoma, or infection (<1% reported incidence)	Minimally invasive, rare complications include bleeding at the puncture site or transient bacteremia (<2%)
Grade of evidence	Validated in clinical practice	Preliminary data; requires validation against HVPG
Clinical utility	Established tool for diagnosing CSPH, assessing TIPS candidacy, and monitoring therapy efficacy	A promising alternative for CSPH diagnosis, with potential applicability in patients where HVPG is contraindicated or for the objective assessment of PSVD
Advantages	Gold reference with robust data	Combines diagnostic and therapeutic capabilities in one session, avoids radiation exposure, and provides direct measurement
Sensitivity and specificity	High sensitivity for sinusoidal portal hypertension; sensitivity > 90%, specificity > 95% for CSPH	Preliminary studies report sensitivity and specificity comparable to HVPG, but data are limited

HVPG: Hepatic venous pressure gradient; EUS-PPG: endoscopic ultrasound-guided portal pressure gradient; CSPH: clinically significant portal hypertension; TIPS: transjugular intrahepatic portosystemic shunt; PT: Prothrombin time; FNA: fine needle aspiration.

## Abbreviations

The following abbreviations are used in this manuscript:

CE-EUS	Contrast-enhanced EUS
EUS	Endoscopic Ultrasound
EUS-PPG	Endoscopic Ultrasound-Guided Portal Pressure Gradient
EUS-LB	Endoscopic Ultrasound-Guided Liver Biopsy
EUS-SWE	Endoscopic Ultrasound-Guided Shear Wave Elastography
HVAT	Hepatic Vein Arrival Time
IPSS	Intrahepatic Portosystemic Shunt
PH	Portal Hypertension
PSVD	Porto-Sinusoidal Vascular Disorder
HVPG	Hepatic Venous Pressure Gradient
TE	Transient Elastography
MRE	Magnetic Resonance Elastography
NASH	Nonalcoholic Steatohepatitis
TIPS	Transjugular Intrahepatic Portosystemic Shunt
LAMS	Lumen-Apposing Metal Stent
AI	Artificial Intelligence
BMI	Body Mass Index
AUROC	Area Under the Receiver Operating Characteristic Curve
